# Functionalizable Sol-Gel Silica Coatings for Corrosion Mitigation

**DOI:** 10.3390/ma11020197

**Published:** 2018-01-26

**Authors:** Jolanta Gąsiorek, Anna Szczurek, Bartosz Babiarczuk, Jerzy Kaleta, Walis Jones, Justyna Krzak

**Affiliations:** 1 Department of Mechanic, Materials Science and Engineering, Faculty of Mechanical Engineering, Wroclaw University of Science and Technology, 25 Smoluchowskiego, 50-370 Wroclaw, Poland; jolanta.gasiorek@pwr.edu.pl (J.G.); anna.szczurek@pwr.edu.pl (A.S.); bartosz.babiarczuk@pwr.edu.pl (B.B.); jerzy.kaleta@pwr.edu.pl (J.K.); 2 BioPharm Enterprises Limited, 65 Gwaun Afan, Cwmafan, Port Talbot SA129EJ, UK; walisjones@yahoo.co.uk

**Keywords:** sol-gel, layers, corrosion inhibitors

## Abstract

Corrosion is constantly a major problem of the world economy in the field of metal products, metal processing and other areas that utilise metals. Previously used compounds utilizing hexavalent chromium were amongst the most effective materials for corrosion protection but regulations have been recently introduced that forbid their use. Consequently, there is a huge drive by engineers, technologists and scientists from different disciplines focused on searching a new, more effective and environmentally-friendly means of corrosion protection. One novel group of materials with the potential to solve metal protection problems are sol-gel thin films, which are increasingly interesting as mitigation corrosion barriers. These environmentally-friendly and easy-to-obtain coatings have the promise to be an effective alternative to hexavalent chromium compounds using for anti-corrosion industrial coatings. In this review the authors present a range of different solutions for slow down the corrosion processes of metallic substrates by using the oxides and doped oxides obtained by the sol-gel method. Examples of techniques used to the sol-gel coating examinations, in terms of anti-corrosion protection, are also presented.

## 1. Introduction

There is a wealth of published data providing information regarding the activation, functionalization and protection of metallic surfaces [1,2,3,4,5,6,7,8,9,10,11]. Such surface modifications are always associated with the need to adapt the surface properties of metallic substrates to a working environment or conditions without a radical change in the metallic material itself. Surface modifications may be applied by different methods such as for surface protection, e.g., protective sol-gel coatings [1,2,5,6,11,12,13], for surface activation, e.g., [14,15] and for surface functionalisation, e.g., hard coatings [3,4,6,10,11,16]. With regards to the types of materials used for surface modification, regardless of the modification process utilised, it is possible to use ceramics, composites, glass, metals, polymers, or, in a more advanced manner, materials such as cermet [17], nanocomposites [18], bioglass [19], oxides [20] or organic-inorganic hybrid materials [21].

One of the rapidly developing methods of surface modification, by which it is possible to obtain ceramics, glass and even composites or hybrid materials, is the sol-gel method. The global market for sol-gel products is evolving and it has been estimated that it will reach more than 3 billion USD before 2020 [22]. The dramatic growth of sol-gel technology results from the combination of many unique and characteristic features in this class of materials, e.g., targeted and made-to-measure, applied on any shape of substrate, low temperature of process, superior homogeneity and purity [23].

Protective coatings obtained by the sol-gel method are widely reported. The most commonly used sol-gel coating matrices are based on silica (SiO_2_) and this review focuses on silica coatings and their modification.

**Figure 1:**
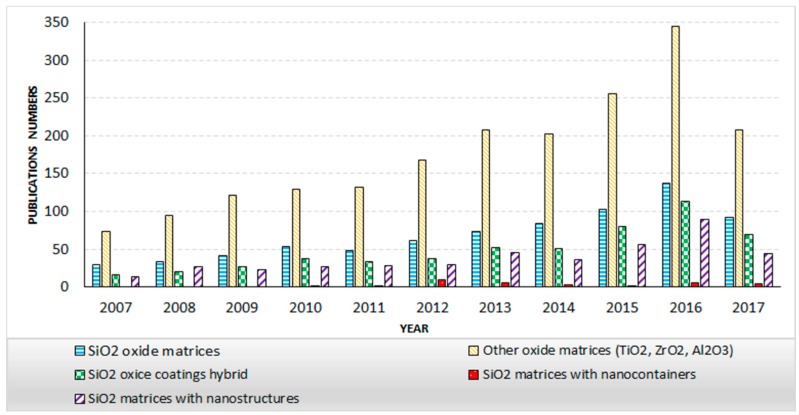
Historical publication data concerning sol-gel matrices for alloys substrates.

The authors analysed the data from the literature alongside their own experience in five groups of SiO_2_ sol-gel coatings, paying special attention to different modifications of SiO_2_ matrices (Figure 1). The following groups were analysed: (1) SiO_2_ oxide matrices on alloy substrates; (2) SiO_2_ matrices with nanocontainers on alloy substrates (3) SiO_2_ matrices with nanostructures on alloy substrates; (4) SiO_2_ hybrid oxide coatings on alloy substrates and (5) other oxide matrices on alloy substrates, such as TiO_2_, ZrO_2_, Al_2_O_3_.

## 2. Sol-Gel Method and the Coatings

### 2.1. Sol-Gel Method

The first notable date for sol-gel chemistry was 1842, when J.J. Ebelmen presented the synthesis of uranium oxide by heating the hydroxide form of uranium [24]. Subsequent works around a hundred years later (R. Roy et al. V. Chiola, et al.) describe the gradual development of sol-gel chemistry [25,26] with a culminating in a key step in the history of the sol-gel field in the 80’s, with an explanation concerning the controlled hydrolysis and condensation of alkoxides (Levene et al. and Dislish et al.) [26]. This was accompanied by another landmark in the chemistry of sol-gel, with the synthesis of a new class of sol-gel materials which were organic-inorganic hybrid materials (OIHMs) [24,27]. These achievements facilitated the expansion of sol-gel chemistry into many fields of science, such as polymer chemistry and ceramics, as well as mitigating corrosion, surface activation, bio-functionalisation and surface engineering, in general [26,28,29].

Sol-gel technology is based on the synthesis of a gel from an organic-inorganic sol by a gelation process [26]. Silicon alkoxides (e.g., tetraethyl orthosilicate (TEOS), tetramethyl orthosilicate (TMOS)) or alkoxides of the transition elements of the periodic table (e.g., zirconium, aluminium, cerium, titanium) are used as precursors in the synthesis of an oxide network in the classical sol-gel method. Sol synthesis proceeds via a sequence of parallel reactions of hydrolysis and condensation (Table 1), resulting in the creation of –M–O–M– oxide bridges between the metal atoms and the formation of molecules of water or alcohol, respectively [30,31,32].

These reactions occur in a suitably selected medium, such as a solvent and in the presence of a suitable catalyst. The choice of solvent has an impact on the structure, thickness and other properties of the resulting sol-gel coatings. Likewise, the choice of a catalyst also has an impact on these properties. The influence of different acidic catalysts on selected oxide structures has been described in our previous paper [23]. Notably, the choice of an acidic catalyst promotes the creation of linear or randomly branched structures [30,31], whereas the use of a basic catalyst leads to the creation of branched structures.

The synthesis of sols via the sol-gel method has to occur during mixing. According to the literature, ultrasonic treatment of the sols promotes the formation of more homogeneous and stable sols, which are very important material and physical characteristics in the context of obtaining durable coatings with protective properties. During the aging of the sol, further condensation and crosslinking of the molecules take place. This leads to a more compact but porous structure, which can have a very important influence on the anticorrosive properties displayed by the sol-gel coatings [33,34].

The next phase in obtain sol-gel coatings is that of the deposition of the sol-gel coating onto the substrate itself and this is discussed in more detail in Section 2.2.1. The final stage of the process involves thermal stabilization. Here again, temperature, conditions can determine the annealing time, with the use of specific heating and cooling gradients determining the properties of the mature sol-gel coatings [1,2,35].

A particularly attractive feature of the sol-gel method is the fact that solid coating materials with a wide application potential can be synthesised and produced at ambient temperature which, apart from being a low-energy process, makes it possible to use thermally-sensitive reactants and for such reactants to preserve their unique features within the sol-gel matrix. Additionally, this method is amenable for coating of large surfaces and of complicated shapes, using cheap, easy-to-use and low-technology equipment. A further advantage of sol-gel synthesis is its use of low-toxicity alternatives to conventional methods requiring the use of hexavalent chromium compounds [1,3,26] This is of particular relevance, in view of the fact that the use of Cr(VI) compounds and materials is no longer permitted in Europe after 21 September 2017, per REACH regulations [36].

All these features combine to making the sol-gel process both eco-friendly and environmentally safe.

### 2.2. Coatings

Today’s science and technology have given rise to many types of protective coatings with anti-corrosion properties, which broadly encompass both metallic and non-metallic coatings. Among the most common non-metallic coatings are inorganic coatings, such as convertible, anodized or ceramic coatings [37]. However, ceramic coatings are becoming increasingly popular within this grouping, especially those obtained by the sol-gel method [1,3,26,28,29].

**Figure 2:**
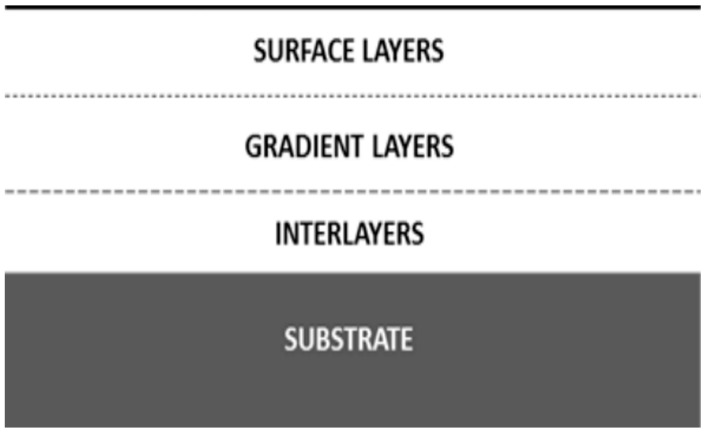
Schematic layout of the respective layers in a coating.

The sol-gel method facilitates the production of coating materials with a wide application potential, with the possibility of achieving flexible control of thin-film morphology. This method is suited for different types of coatings, for example inorganic thin films, hybrid films or coatings doped with different active substances, such as inhibitors of corrosion [1,26]. Moreover, depending on the physicochemical properties of these types of materials, it is possible to obtain combinations of layers which perform various functions in one surface material (Figure 2, Table 2).

#### 2.2.1. Methods of Obtaining Layered Surfaces

There are many techniques available that are suited for coating metallic substrates, such as physical vapour deposition (PVD), chemical vapour deposition (CVD), electrochemical deposition, dip-coating, spin-coating, painting and ultrasonic methods [4,26,35,38,39,40,41] The selection of a suitable coating technique is determined by the properties required of the coating layer. For the sol-gel method, the most frequently used coating techniques are dip-coating and spin-coating [26].

**Figure 3:**
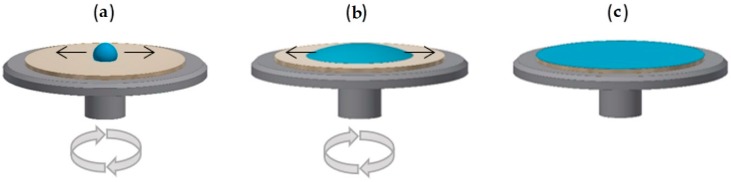
Schematic of the spin-coating technique: (**a**) application of the coating material on the substrate; (**b**) spreading of the coating material over the substrate via rapid rotation or spinning of the substrate; (**c**) resultant thin-film deposition across the substrate.

The application of spin-coating, where the coating material is dripped onto the substrate and is spread by spinning (Figure 3), enables the formation of ultrathin coatings where the layer thickness is controlled by the selection of the rotational speed of the spin-coater and viscosity of the sol [26,38,39] While this method can form very homogenous layers, it is not easily scalable and is only applicable to flat surfaces with finite dimensions. Modifications of the spin-coating technique allow the formation of thin coatings of viscous solutions on hydrophobic substrates. The only limitations to this method are the shape and size of the material to be coated.

**Figure 4:**
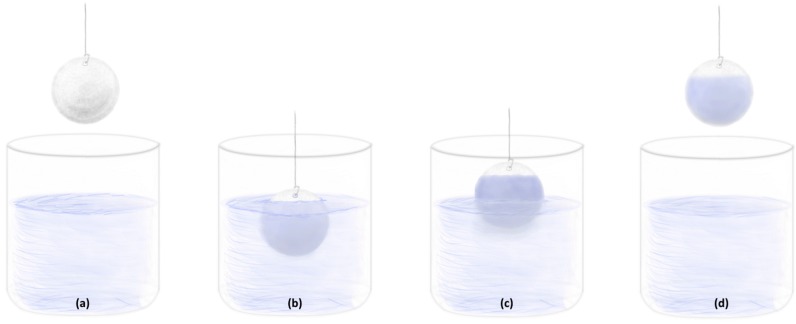
Schematic of the dip-coating technique: (**a**) appropriate presentation of the substrate for the dip-coating procedure; (**b**) immersion of the substrate (optional holding period of the submerged substrate within the coating solution); (**c**) ascent of the substrate from the coating solution; (**d**) fully coated substrate removed from the coating solution.

In the case of multidimensional substrates, dip-coating is considered a better solution since it allows the coating of even very complex substrate shapes (Figure 4). The factor which has a strong influence on the properties of deposited films using this method is the speed of withdrawal of substrates from the coating solutions. For optimal coating, the withdrawal speed is appropriately selected depending upon the viscosity of the coating solution which, in turn, determines the thickness and morphology of the coating [26].

(1) When the liquid viscosity, *η* and withdrawal speed, *U*_0_, are high, such that the curvature of the gravitational meniscus is low, the thickness of the coating layer, *h*_0_ can be defined according to the following equation:
  *h*_0_ = *c*_1_(*ηU*_0_/*ρg*)^1/2^ (1)
  where *ρ* is liquid density, *g* is acceleration of gravity, *c*_1_ is constant.
(2) When the liquid viscosity, *η* and withdrawal speed, *U*_0_, are low, the equation is balanced by considering the liquid-vapour surface tension, *γ_LV_* and the thickness of the coating layer, *h*_0_, can then be defined according to the following equation:
  (2) $$h_{0}=0.94\frac{{\left(\eta U_{0}\right)}^{2/3}}{\gamma_{LV}^{1/6}{\left(\rho q\right)}^{1/2}}$$

Another, though less popular, coating technique is the use of ultrasound. According to the literature this method is useful for obtaining coating layers consisting of particles with regular shape and structure by breaking agglomerates present in the coating solution (e.g., Al_2_O_3_ agglomerate dispersion), resulting in more homogenous coatings characterised by fine-grained microstructure [40]. Ma et al. [42] proved in their work that the control of ultrasound intensity can be used to determine various distributions of elements between a substrate and its coating, which ultimately may have a significant influence on the mechanical properties of the coatings, such as the friction coefficient. Moreover, according to the author, with increasing power of the ultrasound source within a particular frequency range, an improved wettability of the substrate is observed.

#### 2.2.2. Interlayers

Transition layers, also called interlayers, are a group of coating materials which form an interface between a substrate and other adjoining layers (most frequently organic or inorganic-organic hybrid materials). This group of materials need to fulfil certain requirements that encompass good adhesion to the substrate and low porosity, as well as reduced surface irregularity and substrate roughness, otherwise stresses can arise within such deeper layers that could finally lead to cracking [1,13,43].

The most frequently used interlayers, or undercoats, in sol-gel materials are inorganic metal oxides, such as zirconium, silicon, aluminium, cerium or titanium oxides [1,43,44]. An advantage associated with this group of compounds is the large number of Van der Waals bonds that exist between polymer molecules from organic layers and the substrate also. Additionally, these bonds can be transformed into stable covalent bonds using heat treatment.

According to the literature, all oxide-based coatings, such as SiO_2_, ZrO_2_, Al_2_O_3_, TiO_2_, are characterised by low chemical reactivity which can confer substrate protection properties to metallic substrates [11,13,43].

The value of the coefficient of linear thermal expansion for ZrO_2_ (*α*_ZrO_2__ = 11.2 ppm/K) is common to that of numerous other metals (e.g., *α*_Fe_ = 11 ppm/K, *α*_Cu-Ni_ = 12.2 ppm/K, *α*_stali_ = 11–13 ppm/K) and influences the control of the number of fractures resulting from the heat treatment of coatings. Moreover, this oxide is characterised by high hardness [5,43]. Al_2_O_3_ has low electrical conductivity, hence it is frequently used as an insulator. It is also a material that confers protective anticorrosive properties to a metal substrate. Another material with very good corrosion resistance is TiO_2_, which is chemically passive, heat-resistant and shows low electrical conductivity. However, TiO_2_ sols have a relatively low pH and, as a consequence, their direct application to some metal substrates, e.g., magnesium, can be difficult. For this reason, TiO_2_ is usually doped with CeO_2_ [43].

Purely inorganic sol-gel layers, despite their very good adhesiveness to the substrate, show insufficient corrosion protection due to their characteristic mesoporosity and the nanometric, submicron thickness of sol-gel coatings is frequently not high enough to achieve the necessary barrier capacity. Inorganic sol-gel layers are also susceptible to cracking during heat treatment [10]. Hence, inorganic sol-gel thin coatings are insufficient corrosion barriers on their own. For this reason, inorganic matrices are enriched by doping them with functional polymer substances, nanoparticles or corrosion inhibitors, to increase their properties of slow down the corrosion processes. A good example of this approach is inorganic-organic SiO_2_/ZrO_2_ hybrid layers doped with corrosion inhibitors, creating an insoluble interface between a substrate and a gradient coating. The SiO_2_/ZrO_2_ layer is chemically passive and can ensure effective protection, with the corrosion inhibitors introduced into the sol-gel matrix in the form of nanoparticles have a self-protecting capacity [1,43].

#### 2.2.3. Gradient Layers-Organic-Inorganic Hybrid Coating Materials

In this group of materials, the organic component influences the properties of a layer as its thickness increases and improves the resistance of the layer to cracking, while it also increases layer cross-linking of chemical groups, such as epoxy, acrylic or vinyl groups, during the process of additional polymerisation. Doping with organic substances of inorganic matrices, has a positive impact on the barrier-capacity of such layers [1]. Thanks to the inorganic component, layers have typically mechanical properties, such as hardness or more high resistance of abrasions. These mechanical properties can be additionally enhanced by the introduction of nanoparticles, such as montmorillonite [6], or ZrO_2_ [12], to the matrix. Nanoparticles can be introduced into a hybrid matrix in a number of ways, such as in situ, during synthesis, or in the form of a colloidal suspension, including nanometric powders [16].

Another possibility is the introduction of corrosion inhibitors to the hybrid matrix, as they can be introduced in the form of nanoparticles during the synthesis phase [45]. Organic-inorganic materials doped with corrosion inhibitors, such as cerium salts (III), borates, permanganate ions, vanadium compounds, phosphorus compounds, merkaprobenzotiazol, merkaprobenzimidazol, etc. [7,8,46] introduced in the form of nanoparticles, can act as long-term reservoirs of gradually-released compounds which are activated during contact with an aggressive environment. Additionally, the chemical stability of an oxide sol-gel network and the possibility of the gradual dissolution of active compounds, such as corrosion inhibitors, from the oxide network, makes this a perfect material for long-term corrosion protection. Moreover, organic-inorganic hybrids doped with corrosion inhibitors, such as cerium salts (III), have a self-protecting or ‘healing’ effect [1], in that the resultant oxides and cerium hydroxides formed as a result of exposure to the corrosive agent are precipitated at the place of damage and help seal the local area of damage [9].

The group of self-healing materials include hybrid materials utilising micro- or nano-containers in which corrosion inhibitors are encapsulated. This approach has been used in the case of corrosion inhibitors which do not make stable covalent bonds with an oxide hybrid matrix. The capsules themselves are core shell-type particles encasing an active substance, in this case, a corrosion inhibitor. The active substance is released as a result of exposure to corrosion factors or other factors originating from the corrosion processes, such as a change of temperature, pH or specific ion concentration. The active substance is released by the dissolution of the carrier core or through pores in the carrier, depending on the characteristics of the designed carrier [37].

#### 2.2.4. Surface Layers

Surface layers should be characterised not only by their barrier-capacity, in relation to aggressive factors but also by the quality and suitability of their mechanical properties, such as scratch-resistance or the hardness of the surface [16]. Inorganic sol-gel layers exhibit desirable surface properties in this regard. They are hydrophobic and are characterised by limited vapour permeability, which is an important parameter in terms of corrosion protection [10]. Interestingly, layers with evenly distributed pores in their entire volume show poor scratch and abrasion resistance. According to the literature [10,11], surface layers characterised by an established porosity gradient, namely, coatings with a graded rather than a homogenous porosity throughout their entire thickness, show increased scratch resistance. Surfaces layers can be obtained by sol-gel synthesis using, among others, silica precursors [47].

For the purpose of protecting a substrate from corrosion as well as obtaining suitable mechanical resistance of the surface layer, organic-inorganic coatings, doped with corrosion inhibitors obtained by the sol-gel method are commonly used, as they are characterised by high potential in this respect [1,7,8,46]. By careful control of the molar ratios of the inorganic and organic components it is possible to obtain layers which are thicker than in the case of typical inorganic sol-gel films and which are additionally characterised by a high degree of cross-linking and crack resistance (layers with epoxy, vinyl or acrylic groups). Hence, structural properties of film coatings are influenced not only by precursors and solvents used in the synthesis but also by the “exogenous” components introduced. Some corrosion inhibitors show interesting properties as, apart from the inhibiting, anticorrosive action, they can also alter the nature of the sol-gel network that is formed [48]. By way of example, according to X. Zhong et al. the addition of excess cerium can lead to changes in the silica network structure through breakage of silane bonds and their reconstruction as hydroxyl groupings. Such coatings show higher water absorption, evidence of lost barrier properties [49].

## 3. Investigative Techniques

### 3.1. Microscopy

One of the basic tools which serve to characterize sol-gel thin films is microscopy. Depending on the degree of advancement of the particular microscopy technique, it is possible to observe objects to different resolutions. The resolution for optical microscopy is around 200 nm. However, for electron microscopy (EM) it can be as high as 0.05–0.1 nm (this is nearly 4000 times better than that of optical microscopy and nearly 4 million times better than the resolution of the human eye). The undoubted advantage of optical microscopy is the ease of preparation of samples immediately before observation, whereas sample preparation is often more difficult in the case of electron microscopy and requires a lot of experience (TEM, SEM for nonconductive samples). Despite this, an object can be chemically analysed at the same time as EM analysis through the installation of specialized detectors, for example EDS, within the instrumentation.

#### 3.1.1. Light Microscopy (LM)-Metallographic Microscopy (MM)

**Figure 5:**
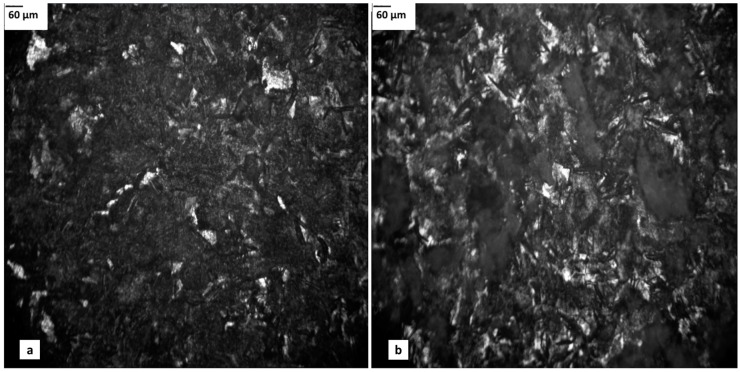
Images taken by a metallographic microscope for steel substrates with phosphate chemical coating: (**a**) after 2 min and (**b**) after 10 min deposition (magnification: ×10).

One of the simplest microscopic techniques to characterise coating materials is optical microscopy, providing a simple way, in terms of continuous analysis for the presence of surface faults, such as detachment of layers from the substrate, delamination, cracks and pitting. However, the method may prove ineffective in the case of very thin films, such as inorganic sol-gel coatings [50]. Another problem that is associated with conventional optical microscopy, where a beam of light is transmitted through the material of interest, is that it is only suited for analysis of transparent materials. Therefore, there is no possibility of taking measurements of non-transparent samples, among others, metals or minerals. This problem has been solved by optical microscopes using reflected light for observation (metallographic), enabling simple microscopic observations of coatings on non-transparent substrates, such as recording layer continuity and surface faults. However, the drawback of this technique is its limited depth-of-field (Figure 5), resulting in issues with image-focusing of contoured surfaces or samples with very rough surfaces. Surfaces which can be observed using MM must be characterised by high light-reflectance. Similar to conventional optical microscopy, the observation of thin transparent films can be difficult [51].

#### 3.1.2. Atomic Force Microscopy (AFM)

**Figure 6:**
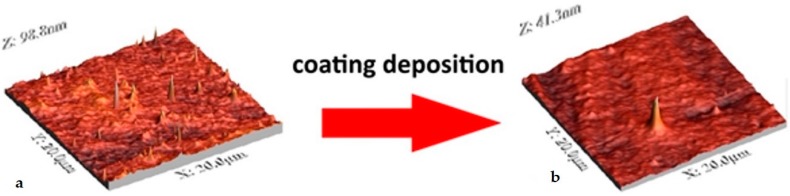
AFM images of poly(ethylene terephthalate) before (**a**) and after (**b**) SiO_2_/TiO_2_ 4-layered coating deposition.

With the limitations of conventional microscopic techniques in analysing metallic substrates and surface coatings, atomic force microscopy has become one of the prime analytical techniques to investigate the morphology of thin transparent films and to obtain 3D-maps of sample surfaces at both micro and nanoscales (Figure 6). The same technique can be used to characterise surface roughness and particle size [50], the homogeneity of substrate coverage by the coating material, or the presence of precipitate in coatings [52].

Indirectly, AFM can also be used to analyse the influence of numerous factors on coating properties. By way of example, Nouri et al. [52] claim that the roughness of thin ZrO_2_ coatings is significantly influenced by thermal stabilisation, observing that increasing temperature during heat treatment was correlated with higher roughness in their AFM measurements. The change in surface roughness was attributed to the change in temperature conditions utilised in the process affecting ZrC_2_ crystal structure. In addition to this, AFM data can also be used to evaluate the Young’s Modulus, on the basis of the Derjaguin-Müller-Toporow model [50].

#### 3.1.3. Scanning Electron Microscopy (SEM)

**Figure 7:**
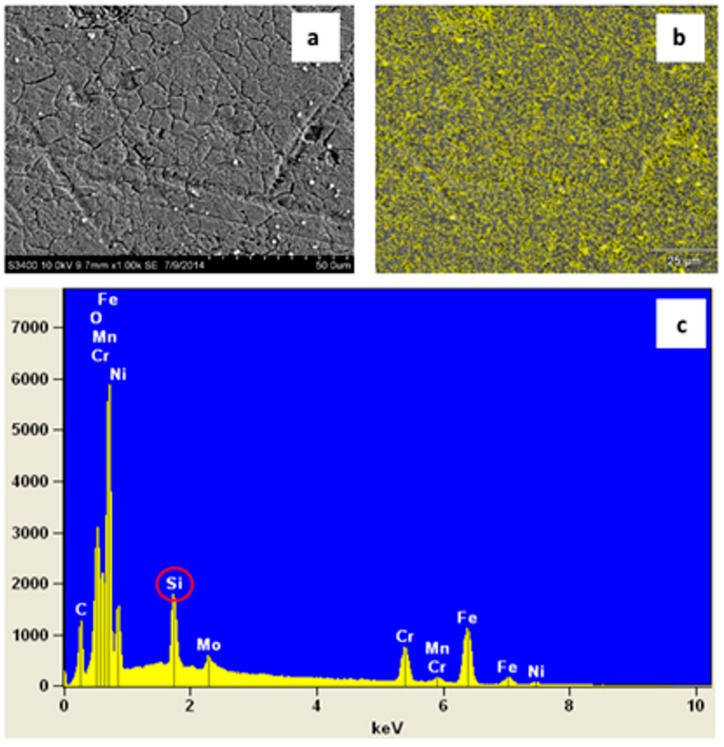
(**a**) Scanning Electron Microscopy (SEM) micrograph of a transparent silica coating on stainless steel (magnification 1000×), (**b**,**c**) EDX analysis shows the presence and uniform distribution of silicon on the substrate surface.

**Figure 8:**
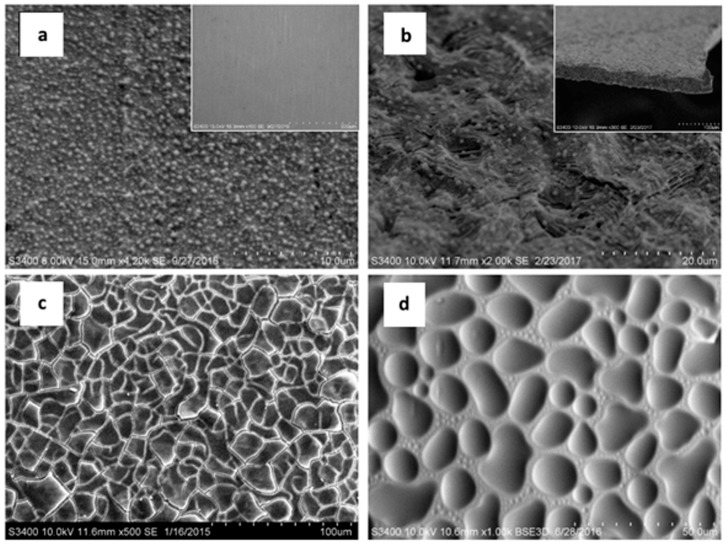
SEM micrographs of silica coatings with different morphologies. (**a**) SiO_2_ coating on the surface of stainless steel: Note the surface appears smooth at low magnification, while a good distribution of visible particles of silica is observable at high magnification. (**b**) SiO_2_ coating on the surface of a titanium alloy substrate after an expansion test: Note it is difficult to see at low magnification, while at high magnification there are visible silica particles. In addition, an interesting property of this coating is its flexibility. (**c**) A cracked SiO_2_ coating on steel. (**d**) SiO_2_ coating with bubbles on the surface of the steel.

Another very useful technique used in surface treatment characterization, as well as in the analysis of the chemical composition of thin films, is scanning electron microscopy (Figure 7). It is a tool which can provide information concerning the potential properties of protective coatings, able to observe morphological changes in coatings, their roughness, homogeneity, transparency, continuity, crystal structure and crystallographic structure defects (Figure 8) [52,53,54,55].

Continuity is one of the most important characteristics of coatings, especially anticorrosive ones and, here, SEM enables the analyses of the whole surface or a particular point. Indirectly, SEM can also be used to draw conclusions on the influence of numerous factors on the structure of coatings, such as heat treatment and processes taking place during the treatment process [52,53,55], the influence of chemical composition on the anticorrosive properties of coating materials [56] and the presence of other phases in a coating [54] and how they impact on the properties of thin film coatings.

A number of detector variants are commonly used, such as secondary electrons (SE), back-scattered electrons (BSE), Energy Dispersive Spectroscopy (EDS) and Wavelength Dispersive Spectroscopy (WDS). SE and BSE detectors enable the imaging of sample surfaces. Secondary electrons are used to analyse the topography and morphology of a surface, while back-scattered electrons are used for the imaging of differences in the composition of multi-phase samples. The EDS detector records X-rays, thus enabling qualitative and semi-quantitative elemental analysis or chemical characterization, while WDS can be used to conduct complete quantitative and qualitative analyses. Moreover, SEM is a technique which is non-destructive for most materials and allows high resolution visualisation of surface structures down to the sub-micron level [57,58].

#### 3.1.4. Transmission Electron Microscopy (TEM)

**Figure 9:**
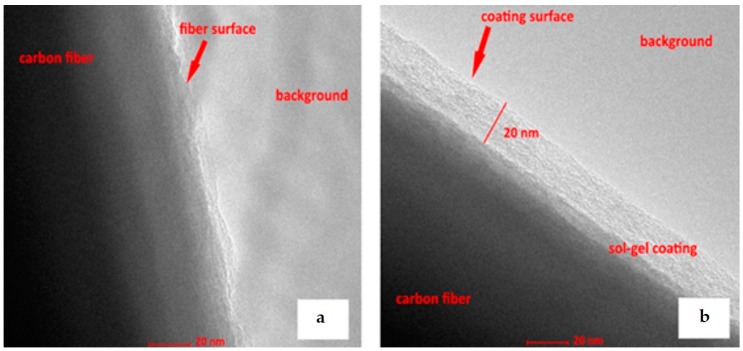
TEM images of: (**a**) uncoated carbon fibre, (**b**) carbon fibre with silica coating.

Transmission electron microscopy enables the sample to be analysed on the basis of the resulting contrast when the samples is overexposed with a suitably shaped electron beam. Thanks to this technique, it is possible to obtain an enlarged image of the irradiated sample created using the detection of electrons interacted by elastic and inelastic scattering (in the case of conventional TEM) with atoms of the sample (which therefore must be every thin). Additionally, other types of interactions between electrons and the sample can be detected, via energy losses or multiple-scattering effects and even electron diffraction. The images can be obtained in different modes, i.e., dark-field, bright-field, phase contrast [59]. TEM allows the determination of morphology of the layer, via the distribution of particles, their shape, grain borders and dislocation. It also enables the observation of individual atoms and defects in their location, as well as the observation of stresses developed in the crystal structure, etc. [37,60,61]. All this means that this apparatus is very useful in determining the characteristics of coatings, such as sol-gels (Figure 9) [37]. TEM has been used to determine the size of the nanometric hybrid particles of modified sol-gel materials [56]. TEM was also used to determine the influence of sol aging time on the structure of sol-gel materials, their degree of crystallinity, amorphousness and the influence of modifiers on the crystal structure. The distinguishing feature of this type of microscopy is the possibility of observing structures to the ultrastructural level and to sub-micron resolution by way of example, Figure 9. illustrates a TEM image of a 20-nanometric thin silica film is presented and also higher magnifications are possible to obtain. Notwithstanding its utility, sample preparation is very difficult, due to the necessity of preparing a sufficiently small and very thin sample for mounting into the instrument.

### 3.2. Spectroscopy

#### 3.2.1. Electrochemical Impedance Spectroscopy (EIS)

Electrochemical impedance spectroscopy (EIS) is one of the main tools enabling the electrochemical properties of coatings to be characterised, including parameters characterising corrosion processes taking place at the surface of metallic materials. EIS can be used to determine the barrier capacity of a surface layer, its electrical resistance and capacity and even its thickness can be determined at the nanometric scale [56,62]. It is a tool that can be used to determine the transition from the initial passive state of the coating/substrate system to the final active state and can also establish under what conditions a given coating loses its protective properties [57,63].

EIS is an ideal tool for investigating the kinetics of corrosion processes, providing dynamic information on reaction mechanisms at the border of the metal substrate and within the coating phases and to measure changes in these mechanisms with time [50,55,56,63] By way of example, Barranco et al. [64] used EIS to characterise the anticorrosive properties of hybrid sol-gel coatings over a range of exposure times extending from 90 min to 7 days in a given medium (0.5 M Na_2_SO_4_), demonstrating that organic-inorganic hybrids showed better barrier capacity, thanks to the development of thicker and crack-resistant films, in comparison with films obtained from purely inorganic materials. The loss of coating stability and, hence, the process of surface degradation, was observed as a decrease in the module impedance value. However, the high impedance value suggests that the coating was not fractured on a particular surface, i.e., it did not undergo a degradation process. During the measurements, such values as time constants, which indicate faults and significant porosity, are obtained. Nouri and co-workers [52] used EIS to observe that ceramic coatings efficiently limited the flow of ions to the substrate and that their transfer was possible only via the occurrence of pores and cracks in the coating. The same method can be used to test the influence of other factors, such as an increase in temperature during thermal stabilisation [64] or the influence of chemical composition [54] on the protective properties of coatings. EIS is therefore considered as a key tool for determining long-term anticorrosive properties of films, including thin sol-gel films.

#### 3.2.2. Raman Spectroscopy and Fourier Transform Infrared Spectroscopy (FTIR)

**Figure 10:**
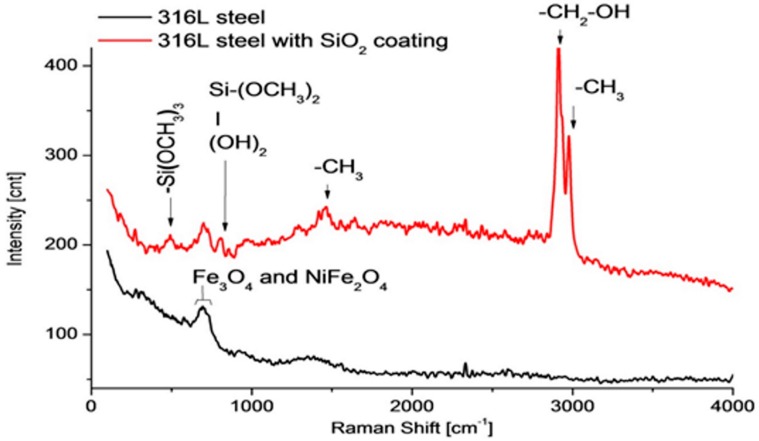
Raman spectra of 316L steel before (black spectrum) and after (red spectrum) SiO_2_ coating deposition.

Raman spectroscopy is a contactless, non-destructive and non-invasive technique that can be used to identify the vibrational characteristics of molecules. Moreover, it enables the recording of far-infrared spectra, where numerous bands that are characteristic of specific inorganic compounds can be observed (Figure 10), including also the characteristics of such groups as SiO_2_, Al_2_O_3_ and ZrO_2_. The method can be used to identify the chemical composition of a sample and the distribution of chemical groups on a surface (mapping) [65,66]. This allows also the definition of layer reactivity as well as the chemical nature of a layer [37]. In terms of corrosion-resistant materials, Raman spectroscopy can identify corrosion products [65], protective materials and can also be used to detect stresses generated in materials [65] and to investigate interactions between sample components.

A more developed version of infrared spectroscopy (IR), in which a sample is analysed using a monochromatic beam, is Fourier Transform Infrared Spectroscopy (FITR), which uses a full-range IR beam. FTIR is a quicker, more sensitive and more accurate method than classical IR. This is a technique which complements Raman spectroscopy, due to the possibility of analysing materials which cannot be analysed optimally by Raman spectroscopy. However, in parallel with Raman spectroscopy, FTIR can also be used to identify functional groups and characteristic chemical bonds in particular materials, which can provide information concerning the network structure within the material [53,56], along with its faults [64], the phase transitions of the components of coating materials and the influence of numerous factors on a coating, such as heat treatment [52]. FTIR allows also the investigation of the influence of other additives, e.g., pigments, on bonds generated in a coating material [10]. This, in turn, may provide information on the reaction-mechanisms that take place within coating materials when various components are introduced in their structure, such as pigments, corrosion inhibitors, or organic precursors.

#### 3.2.3. X-ray Photoelectron Spectroscopy (XPS)

**Figure 11:**
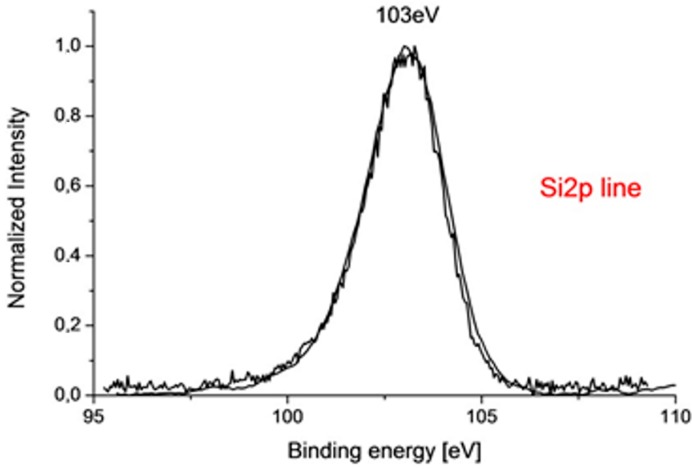
High-resolution spectra of Si2p line in SiO_2_/TiO_2_ hybrid coating.

Thanks to its high sensitivity, X-ray Photoelectron Spectroscopy enables both qualitative and quantitative chemical analysis of thin-films whose thicknesses are in the submicron or nanometric scales. XPS spectra can identify chemical bonds between elements which, in turn, can provide an indication of the reactivity of the analysed material [67] or the degree of components reacting. The technique can also be used to determine the degree of oxidation of the components in the analysed material [66] and to analyse the anti-corrosive nature of sol-gel coatings [48]. XPS is able to provide a quantitative analysis of the elements present in a coating material and characterise the distribution of the elements or chemical groups in a coating material, along with an indication of the types of chemical bonds that are present within the layer [67]. By way of example, in Figure 11 a high-resolution spectrum of Si2p line in an SiO_2_/TiO_2_ hybrid coating is presented. This spectrum indicates bonding of Si in SiO_2_. The method can also generate a depth-profile by argon-etching of appropriate layers within the surface. XPS can be used to conduct chemical analysis at a particular point as well as across a selected area on the sample surface.

## 4. Conclusions

### 4.1. In General

Recently, sol-gel technology has been an intensively researched and developed method for protecting metallic substrates from corrosion and are showing encouraging developments applicable across a wide range of end-user applications. It is a method with enormous potential, applicable not only within the laboratory environment but also, importantly, on an industrial scale, where its commercial potential can be exploited to the full [22]. Numerous studies devoted to this issue report various modifications related to coating methods for obtaining functional and durable sol-gel coatings [2,3,25,37,38,39,56,67] synthesis procedures, including changes in the composition [40,42] and processing of coatings [37]. Sol-gel coatings are characterised by low thickness, which poses particular requirements to researchers investigating the morphology and properties of these coatings.

Currently, in the research on the protective properties of sol-gel coatings, the most frequently used techniques encompass microscopy and spectroscopy. The continuous progress in the development of these techniques enables the analysis of increasingly smaller objects with a simultaneous higher level of accuracy and definition of measurements. This brief review offers a clear and concise summary covering anticorrosive sol-gel coatings and the most frequently used measurement techniques associated with their production and analysis. The article discusses the structure of sol-gel coatings and the requirements which have to be met by particular layers, so that they can protect metallic substrates used not only under laboratory conditions but can be translated into functional and durable materials in the wider working environment. The most important up-to-date modifications of these layers in terms of obtaining materials characterised by increased corrosion resistance are also described.

It is worth emphasising that an increasingly frequent solution in the context of the corrosion protection of metallic substrates is the use of various materials in one sol-gel coating, either as inorganic [4,32,37,40,42], organic [30] or organic-inorganic hybrids [1,9,43]. Finally, the resulting materials are hybrid and/or layered materials and/or nanocomposites which are characterised by different properties. However, when jointly applied, they result in coatings with the required characteristics.

### 4.2. In Detail

The review distinguishes two areas which need to be developed more carefully in labs and on the prototyping stage:

1. Obtaining anticorrosive coatings using organic-inorganic hybrid materials.
  This area is related to the still poorly-recognized processes that occur during gel formation, namely, the chemical and physical processes that take place between organic and inorganic components that define the structural characteristics of the layer, (such as the spacing or interpenetration between polymer chains and inorganic matrices, or the occurrence of phase-separation processes involving the formation of systems which can be partially mixed with water during gelling [55]), which can have a significant influence on the final properties of coatings.
2. Decreasing the porosity of protective coatings obtained with the sol-gel method.
  Generally, most sol-gel materials are described as mesoporous. Porosity can be modified by the use of nanoparticle/nanofiller doping, such as montmorillonite [6], ZrO_2_ [12], as well as active organic or inorganic compounds, such as corrosion inhibitors (cerium(III) compounds [62]) and, also sometimes, precursors with nonhydrolysable organic groups, e.g., methyl or ethyl groups. Moreover, by appropriate control of the aging-time of hydrolysates (e.g., 24 h), it is possible to thicken the structure using progressing sol-gel reactions (discussed in chapter 2.1). In the case of sol-gel coating materials, the right selection of substrates (which determine resultant components) and the arrangement of layers, frequently allows the separation of the substrate from the working environment at the required level in actual real-life scenarios.

In this review, excellent features of the sol-gel based materials and features of technique itself, which allow us to apply in the broadly understood corrosion protection, have been presented and they can be summarized as follows:

- ability to dedicate the composition, both to the selected substrate and to the chosen environment by applying multilayer coatings
- chemical inertion of inorganic oxide matrices and very good adhesion to the metallic substrates
- easy doping or modification of sol-gel matrices
- possibility of modifying the parameters of synthesis and surface or volume functionalization, results in sealing the network and thus increasing barrier properties

However, different problems have been also discussed and the obvious summary is, that solution for certain limitations should be provided to allow a wider application of sol-gel protective materials. What’s more, work on limitation is carried out in laboratories around the world. In short:

- application of sol-gel silica layers to elements working in an aqueous or alkaline environment is limited and forces the progress of development of stable coating materials in various environments
- work on the synthesis of highly corrosion-resistant sol-gel layers with high hardness and abrasion resistance are still in progress
- one of the sol-gel synthesis routes is based on acid catalysis, which translates into low pH of sols, which in turn limit their application to the acids sensitive substrates, e.g., magnesium
- there is also a problem of using some sol-gel organic-inorganic hybrids directly on the surface of metallic materials, which results from the separation of the organic phase in the inorganic matrix.

Finally, it should be underlined that the corrosion phenomenon can be severely limited by the use of functionalized sol-gel silica materials.

## Figures and Tables

**Table 1:** Sol Synthesis Reaction.

Reaction	Formula
Hydrolysis reaction	M–OR + H_2_O → M–OH + ROH
Condensation reactions	
Water condensation	M–OH + HO–M → M–O–M + H_2_O
Alcohol condensation	M–OR + HO–M → M–O–M + ROH

**Table 2:** A summary of the types of coating materials.

Type of Layer	Key Features	Examples
Inert Layers	Low porosity	SiO_2_ [1], ZrO_2_ [5,43], Al_2_O_3_ [43], TiO_2_ [43]
	Substrate adhesion	
	Reduced roughness	
Gradient Layers	Low porosity	Oxide matrices with GPTMS [6]/VETO [49]/PMMA [37] doped by corrosion inhibitors [7,8,46,49], nanostructures [1,6,12,45] or nanocontainers [37]
	Crack resistance	
	Inhibition of corrosion	
Surface Layers	Low porosity	ZrO_2_ [1,10], SiO_2_-GPTMS-VETO doped by cerium nitrate [49]
	Mechanical resistance	
